# Determinants of functioning and health-related quality of life after vestibular stroke

**DOI:** 10.3389/fneur.2022.957283

**Published:** 2022-09-08

**Authors:** Franziska Schuhbeck, Ralf Strobl, Julian Conrad, Ken Möhwald, Patricia Jaufenthaler, Klaus Jahn, Marianne Dieterich, Eva Grill, Andreas Zwergal

**Affiliations:** ^1^German Center for Vertigo and Balance Disorders, DSGZ, LMU Hospital, Ludwig-Maximilians Universität Munich, Munich, Germany; ^2^Department of Neurology, LMU Hospital, Ludwig-Maximilians Universität Munich, Munich, Germany; ^3^Institute for Medical Information Processing, Biometry, and Epidemiology, Ludwig-Maximilians Universität Munich, Munich, Germany; ^4^Schön Clinic Bad Aibling, Department of Neurology, Bad Aibling, Germany; ^5^Munich Cluster for Systems Neurology, SyNergy, Munich, Germany; ^6^Munich Center for Health Sciences, Ludwig-Maximilians Universität Munich, Munich, Germany

**Keywords:** vertigo, vestibular disorders, stroke, quality of life, outcome prediction

## Abstract

**Background:**

Stroke accounts for 5–10% of all presentations with acute vertigo and dizziness. The objective of the current study was to examine determinants of long-term functioning and health-related quality of life (HRQoL) in a patient cohort with vestibular stroke.

**Methods:**

Thirty-six patients (mean age: 66.1 years, 39% female) with an MRI-proven vestibular stroke were followed prospectively (mean time: 30.2 months) in the context of the EMVERT (EMergency VERTigo) cohort study at the Ludwig-Maximilians Universität, Munich. The following scores were obtained once in the acute stage (<24 h of symptom onset) and once during long-term follow-up (preferably >1 year after stroke): European Quality of Life Scale-five dimensions-five levels questionnaire (EQ-5D-5L) and Visual Analog Scale (EQ-VAS) for HRQoL, Dizziness Handicap Inventory (DHI) for symptom severity, and modified Rankin Scale (mRS) for general functioning and disability. Anxiety state and trait were evaluated by STAI-S/STAI-T, and depression was evaluated by the Patient Health Questionnaire-9 (PHQ-9). Voxel-based lesion mapping was applied in normalized MRIs to analyze stroke volume and localization. Multiple linear regression models were calculated to determine predictors of functional outcome (DHI, EQ-VAS at follow-up).

**Results:**

Mean DHI scores improved significantly from 45.0 in the acute stage to 18.1 at follow-up (*p* < 0.001), and mean mRS improved from 2.1 to 1.1 (*p* < 0.001). Mean HRQoL (EQ-5D-5L index/EQ-VAS) changed from 0.69/58.8 to 0.83/65.2 (*p* = 0.01/*p* = 0.11). Multiple linear regression models identified higher scores of STAI-T and DHI at the time of acute vestibular stroke and larger stroke volume as significant predictors for higher DHI at follow-up assessment. The effect of STAI-T was additionally enhanced in women. There was a significant effect of patient age on EQ-VAS, but not DHI during follow-up.

**Conclusion:**

The average functional outcome of strokes with the chief complaint of vertigo and dizziness is favorable. The most relevant predictors for individual outcomes are the personal anxiety trait (especially in combination with the female sex), the initial symptom intensity, and lesion volume. These factors should be considered for therapeutic decisions both in the acute stage of stroke and during subsequent rehabilitation.

## Introduction

Acute stroke is responsible for 5% of all disability-adjusted life years (1). However, data on functional outcomes are almost exclusively available for patients with anterior circulation stroke, while prognostic markers for disability and functioning are largely missing for posterior circulation strokes (2). Frequently observed chief complaints associated with posterior circulation stroke, such as acute vertigo, dizziness, double vision, or gait instability, are underrepresented in clinical tools for stroke assessment, such as the National Institutes of Health Stroke Scale (NIHSS) (3). In consequence, there is no consensus, which symptoms should prompt a more extensive therapy during the acute stage (e.g., intravenous thrombolysis) and which patients should receive more intense rehabilitation on the course (4).

In vestibular disorders, several factors contribute to symptom severity, health-related quality of life (HRQoL), and psychological comorbidity (5–7). Generally, subjective symptoms and HRQoL tend to correlate with objective tests of semicircular canal function in acute peripheral and central vestibular disorders only (8), while there is no direct relationship between labyrinthine function and symptom intensity in their chronic stage (9). Long-term adaptation to deficits in vestibular processing seems to depend rather on the time course of vestibular symptoms (recurrent vs. chronic), the character traits of the patient (e.g., anxiety), as well as coping and resiliance mechanisms (10). Episodic vestibular syndromes such as vestibular migraine or Menière's disease are most frequently associated with anxiety and depression (11, 12), while patients with chronic unilateral or bilateral peripheral vestibulopathies do not have more psychiatric comorbidities than healthy controls (13). Signs of vestibular imbalance in central vestibular lesions (such as deviation of the subjective visual vertical) recover with a similar time course than in unilateral peripheral vestibulopathies (14), but it remains unclear, if this translates to an improvement of HRQoL and functioning.

In this study, we aimed to evaluate the trajectories and determinants of long-term functioning and HRQoL in a well-characterized cohort of patients with acute posterior circulation stroke, presenting with the chief complaints of acute vertigo, dizziness, double vision, or imbalance, based on a follow-up assessment. We hypothesized that the most relevant factors for the outcome will be the symptom intensity during the acute stroke stage, age, sex, localization, and volume of the lesion, as well as indicators of accompanying anxiety and depression.

## Methods

### Patient characteristics and study protocol

This prospective long-term follow-up study included a cohort of 36 patients (age: 66.1 years, sd: 12.0 years, 38.9% women), who initially presented to the Emergency Department (ED) of the LMU Hospital, Munich, with the chief complaints of acute vertigo, dizziness, double vision, or postural imbalance (symptom onset <24 h, symptom duration >10 min), and were subsequently diagnosed to have an acute brain stem-cerebellar stroke in MRI. Patients were recruited prospectively in the scope of the EMERT (EMergency VERTigo) trial (15). All patients received a *standardized and comprehensive investigation in the ED* including history taking, clinical neurological and neuro-otological examination, video-oculographic assessment (EyeSeeCam, Fürstenfeldbruck, Germany) of vestibular and ocular motor functions (e.g., video head impulse test, spontaneous nystagmus, smooth pursuit, gaze holding, and saccades), mobile posturography, bucket test for subjective visual vertical (SVV, normal range 0 ± 2.5°) (16), as well as scales and scores for symptom severity (Dizziness Handicap Inventory, DHI), disability and functioning (modified Ranking Scale, mRS), and HRQoL (European Quality of Life Scale-five dimensions-five levels, EQ-5D-5L; European Quality of Life Scale—Visual Analog Scale, EQ-VAS). *A follow-up assessment* was done once after a mean time of 30.2 months (sd: 11.9 months) by standardized patient interviews including DHI, mRS, EQ-5D-5L, EQ-VAS, scales for anxiety (State-Trait-Anxiety Inventory—State and Trait: STAI-T, STAI-S), and depression (Patient-Health-Questionaire-9, PHQ-9). Furthermore, clinical neurological and neuro-otological assessments of vestibular, ocular motor, and postural functions were added. The long timespan for follow-up of preferably >1 year after stroke was chosen based on the assumption that a stable functional status of recovery should have been reached.

### Protocol approval and patient consent

The study was approved by the Ethics Committee of the University of Munich on 02/23/2015 (57-15) and was conducted according to the Guideline for Good Clinical Practice, the Federal Data Protecting Act, and the Helsinki Declaration of the World Medical Association. All subjects gave their informed, written consent to participate in the study. The study was listed in the German Clinical Trial Registry under the ID DRKS00008992 and the Universal Trial Number ID U1111-1172-8719.

### Scores and scales

We collected, standardized, and established scores and scales to assess HRQoL, functioning, symptom severity, and psychiatric comorbidity following vestibular stroke.

The *EQ-5D-5L questionnaire* consists of five questions, called dimensions (i.e., mobility, self-care, usual activities, pain/discomfort, and anxiety/depression), each with five answer choices (1–5), called levels. The result is reported as a five-digit number. In the present study, the EQ-5D-5L index was calculated from these figures using the German value set as a reference (−0.661 worst health status, 1 best health status) (17).

The EQ-5D-5L questionnaire also includes a visual analog scale, the *EQ-VAS*, which is used to determine the patients' current perceived health status. The EQ-VAS is a vertical scale with values ranging from 0 to 100, where 0 represents the worst and 100 represents the best state of health that the patient can imagine.

Patients' functionality and disability was evaluated using the *mRS*. The mRS ranges from 0 (no symptoms) to 6 (death) and describes the degree of patients' impairment and disability after stroke. The mRS can also be used to assess the outcome. In some studies, a favorable course is usually defined for values from 0 to 2 (18, 19).

The *DHI* was applied to rate the patients' subjective symptom severity due to vertigo and dizziness. The DHI is composed of 25 questions that assess the functional, emotional, and physical impact of vertigo and dizziness on the patient. Scores ranging from 0 (no impairment due to dizziness) to 100 (significant subjective impairment) are possible (20).

In the follow-up interview, anxiety and depression as frequent psychiatric comorbidities were specifically assessed. The STAI questionnaire, consisting of the *STAI-S* and *STAI-T*, each with 20 statements, was used to quantify anxiety. The STAI-S evaluates the patients' current level of anxiety, while the STAI-T indicates the extent to which anxiety is part of the patients' personality traits and is therefore thought to be a stable condition (21). Scores between 20 and 80 are possible in each case, with higher scores indicating increased anxiety. The *PHQ-9* was applied to identify depression in patients. The PHQ-9 contains nine questions based on the Diagnostic and Statistical Manual of Mental Disorders (DSM-IV) criteria for depressive disorders. The patient indicates how often various symptoms have occurred in the past 2 weeks (from 0: “not at all” to 3: “nearly every day”). A depressive disorder is considered, if at least two questions were answered with “more than half of the days” (=2), and depression or anhedonia is one of the symptoms mentioned. In general, a total score between 0 and 27 points is possible and provides information about the severity of a depressive disorder (22, 23). A *specially customized questionnaire* was developed to further evaluate the persistence of vertigo and dizziness, symptomatic days, as well as further strokes during the time to follow-up.

### Magnetic resonance imaging and lesion mapping

A standardized MRI was performed within the first 7 days after stroke (mean 2.2 days, sd: 2.5 days), including whole brain and brain stem diffusion-weighted images (DWI), fine-slice 3 mm fluid-attenuated inversion recovery (FLAIR), T2, T2^*^, and 3D-T1, time-of-flight (TOF) angiography. Statistical parametric mapping (SPM) was used to determine lesion volume in voxels. Lesions were delineated on acute phase brain stem T2-weighted or brain stem DWI sequences using MRIcron. All lesion maps were then normalized into 1 × 1 × 1 mm^3^ MNI space using the Clinical Toolbox in SPM for visualization (24). In addition, stroke lesions were assigned to vascular territories by two expert neuro-radiologists. Furthermore, the Fazekas score for micro-vascular lesions was assessed on FLAIR sequences. The Fazekas score consists of 4 levels (range 0–3), describing the extent of hyperintensity in the periventricular and deep white matter (25).

### Statistical analysis

All data were collected and organized by a REDCap (Research Electronic Data Capture) database. This software platform enabled error-free data entry and export to SPSS, as well as validation, quality control, and secure storage of the data (26).

Statistical analysis was carried out with SPSS (IBM SPSS Statistics, version 27.0.1.0). In the descriptive analysis, means, standard deviations (sd), variance, and sum scores of the questionnaires and variables were calculated. Additionally, absolute and relative frequencies of different variables were determined. Depending on the scale level, linear correlations were computed using the Bravais-Pearson, Spearman or Eta coefficient, or the Chi^2^ test. *T*-tests for dependent samples were performed to analyze whether the progression parameters (DHI, mRS, EQ5D-5L, EQ-VAS) had changed over time. Independent sample *t*-tests were used to investigate whether distinct subgroups of the study population differed in their progression parameters.

Multiple linear regression models were calculated to analyze the factors predicting the long-term outcome of vestibular stroke patients. Two variables were selected to describe the outcome: Symptom-related outcome was represented by the DHI at follow-up and general health outcome by the EQ-VAS at follow-up. For both variables, a separate model was calculated. Patient-specific and lesion-specific variables were selected as predictors to comprehensively investigate possible factors influencing outcome: patient age at the time of stroke, gender, STAI-T, and DHI in the acute stage as patient-specific variables, and lesion volume measured in voxels as a lesion-specific variable.

## Results

### Clinical and imaging characteristics of the study cohort

Patients of the study cohort most frequently reported vertigo, dizziness, and double vision as their chief complaints during the acute stage of symptoms (Supplementary Table 1). Upon clinical neurological and neuro-otological exam, 21 patients had central ocular motor signs (such as saccadic smooth pursuit, direction-changing gaze-evoked nystagmus, and cross-coupling during head shaking), 19 patients had signs of vestibular asymmetry (such as spontaneous nystagmus, SVV tilt, and skew deviation), 16 patients had gait imbalance, and 5 patients had mild limb ataxia. Most patients had an extensive cardiovascular risk profile, with the most common factors being arterial hypertension, hypercholesterolemia, nicotin abuse, and a positive family history of cardiovascular disease. In the acute stage of symptoms, DHI was 45.0 ± 24.2, mRS was 2.1 ± 1.2, EQ-5D-5L index was 0.69 ± 0.3, and EQ-VAS was 58.8 ± 19.1 (Table 1).

**Table 1:** Health-related quality of life (HRQoL), functioning, and symptom severity at acute stage and follow-up.

	**Acute stage**	**Follow-up**	***t*-test**
**DHI**	45.0 ± 25.2	18.1 ± 24.8	*p < * 0.001, *t =* −5.4
**mRS**	2.1 ± 1.2	1.1 ± 1.3	*p < * 0.001, *t =* −4.2
**EQ-5D-5L index**	0.69 ± 0.3	0.83 ± 0.2	*p =* 0.014, *t =* 2.6
**EQ-VAS**	58.8 ± 19.1	65.2 ± 18.0	*p =* 0.112, *t =* 1.6

DHI, Dizziness Handicap Inventory; EQ-5D-5L, European Quality of Life-5 Dimensions-5 Levels questionnaire; EQ-VAS, European Quality of Life Scale—Visual Analog Scale; mRS, modified Rankin Scale.

**Figure 1:**
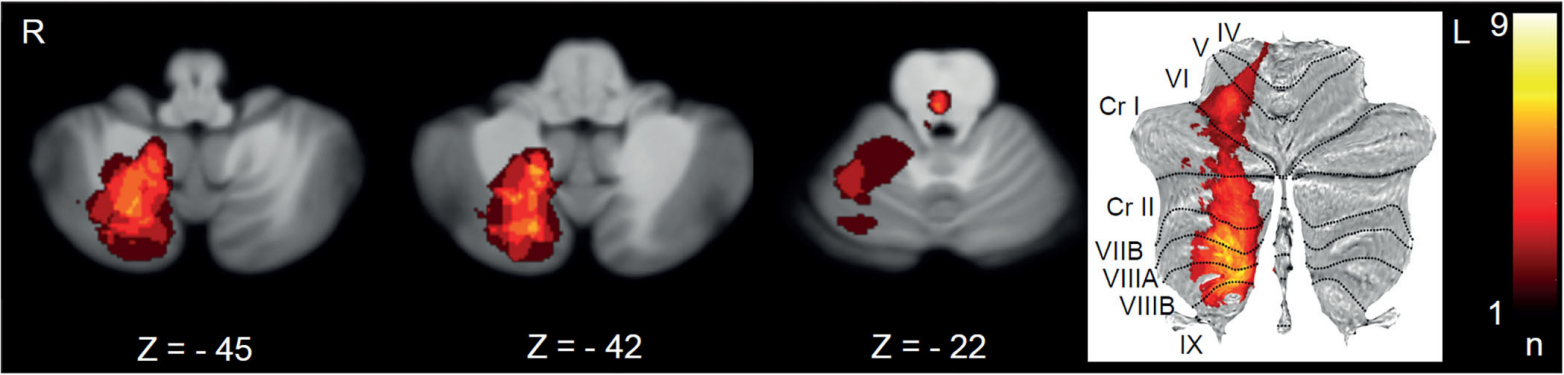
Lesion mapping of all strokes with the chief complaint of vertigo, dizziness, or double vision depicted on transverse sections (*Z*-scores in MNI space) and as a surface plot. L, left; R, right.

Stroke lesions were mapped to the medial cerebellum and brain stem (Figure 1). The mean lesion volume in 1 mm isovoxels was 5,298 (sd: 9,244). In the cerebellum, most lesions were localized in the territories of the posterior inferior cerebellar artery (PICA) (30.6%) and the superior cerebellar artery (SCA) (8.3%). The lobules VIIIA/B (biventer lobule: 27.8%), lobules VII (inferior semilunar lobule: 19.4%; superior semilunar lobule: 11.1%), vermal lobules IX and X (uvula/nodulus: 8.3%), and lobules IV and V (anterior and posterior quandrangular lobule: 8.3%) were affected predominantly (Figure 1). Brain stem lesions affected the midbrain (27.8%), pons (19.4%), and medulla (5.6%) most frequently. The most prevalent etiologies for posterior circulation stroke were arterio-arterial embolism (44.4%) and cardiac embolism (30.6%). Fazekas score for cerebral microangiopathy was 1.1 ± 1.0.

### Outcome parameters during follow-up

Compared to the acute stage, during follow-up (after a mean time of 30.2 months), DHI had significantly improved to 18.1 ± 24.8 (*p* < 0.001), mRS had improved to 1.1 ± 1.3 (*p* < 0.001), and EQ-5D-5L had improved to 0.83 ± 0.2 (*p* = 0.014), while EQ-VAS remained low (*p* = 0.112) (Table 1). The scales for anxiety at follow-up were 36.4 ± 11.2 (STAI-T) and 34.2 ± 12.1 (STAI-S). The depression score PHQ-9 was 3.2 ± 3.9. Two of the 36 enrolled patients had died, seven patients had another stroke (after a mean time of 10.7 ± 11.4 months), and nine patients had persistent dizziness or imbalance. These patients reported to have 13.9 ± 13.8 days per month, where they perceived balance-related symptoms.

Clinical follow-up assessment indicated mild central ocular motor signs in 8 patients, some degree of gait or limb ataxia in 6 patients, and signs of enduring vestibular asymmetry (deviation of SVV, provocation nystagmus) in only 4 of 34 patients.

### Influencing factors on functional outcome

A multiple linear regression model with the dependent variable DHI at follow-up showed an independent and significant effect of an anxiety trait measured by STAI-T (*p* < 0.001), especially in combination with female gender (*p* = 0.01), of lesion volume (*p* = 0.04), and symptom severity (represented by DHI) in the acute stroke stage (*p* = 0.01), while age was irrelevant for the outcome (Table 2). When EQ-VAS at follow-up was chosen as an outcome variable for overall health status, only STAI-T (*p* = 0.01) and DHI during the acute stage (*p* = 0.02) persisted to be significant influencing factors, while lesion volume was not relevant (*p* = 0.18). In this model, age was an independent factor for outcome prediction (*p* = 0.03) (Table 3).

**Table 2:** Multiple linear regression model with DHI at follow-up as dependent variable and outcome parameter.

**Model**	**Unstandardized**		**Standardized**	**Significance**
**variables**	**coefficients**		**coefficients**	**level**
	**B**	**Standard**.	**Beta**	
		**error**		
(Constant)	−97.45	31.98		0.01
Sex	−5.59	8.37	−0.11	0.51
Age	0.64	0.33	0.36	0.06
STAI-T	1.31	0.38	0.61	0.00
Lesion volume	0.92	0.42	0.33	0.04
DHI acute stage	0.49	0.18	0.52	0.01
STAI-T_Sex	2.31	0.75	0.54	0.01

Lesion volume: in voxels divided by factor 1,000; STAI-T_Sex: interaction variable of STAI-T and female sex. R^2^ = 0.60; Adjusted R^2^ = 0.48; Significance level < 0.05.

**Table 3:** Multiple linear regression model with EQ-VAS at follow-up as dependent variable and outcome parameter.

**Model**	**Unstandardized**		**Standardized**	**Significance**
**variables**	**coefficients**		**coefficients**	**level**
	**B**	**Standard**.	**Beta**	
		**error**		
(Constant)	153.07	26.13		0.00
Sex	5.85	6.84	0.15	0.40
Age	−0.64	0.27	−0.47	0.03
STAI-T	−0.87	0.31	−0.53	0.01
Lesion volume	0.48	0.35	0.22	0.18
DHI acute stage	−0.38	0.14	−0.51	0.02
STAI-T_Sex	0.11	0.61	0.03	0.87

Lesion volume: in voxels divided by factor 1,000; STAI-T_Sex: interaction variable of STAI-T and female sex. R^2^ = 0.56; Adjusted R^2^ = 0.43; Significance level < 0.05.

## Discussion

The major findings of this longitudinal cohort study in patients with vestibular stroke were the following: (1) The overall prognosis of vestibular stroke was favorable. Scores for HRQoL, functioning and symptom intensity indicated a relevant long-term improvement on a group level. (2) The most important prognostic markers for enduring symptoms were patient-related factors, such as a higher degree of individual anxiety trait, especially combined with female gender, lesion-related factors, namely, stroke volume, and symptom-related factors, i.e., symptom severity in the acute stroke stage. (3) Worse subjective overall health status after vestibular stroke was related to older age, more trait anxiety, and more severe acute vertigo/dizziness symptoms. These findings emphasize the need for a multifaceted evaluation of patients with posterior circulation stroke (including psychological traits), in order to better estimate their functional outcome, and tailor therapeutic decisions (in the acute stage and during rehabilitation) to individual risk. In the following discussion, we will specifically address the importance of symptom-, patient-, and lesion-related features for functional outcomes in vestibular stroke.

### Relevance of acute vestibular symptoms for functional outcome

The extent of acute symptoms of a vestibular stroke may greatly vary across patients (27). A recent study showed that patients with acute central vestibular disorders on average tend to have less vertigo-related symptoms than patients with acute peripheral vestibulopathies (8). However, until now it remained unclear, if the acute symptom load may also predict the long-term functional outcome in vestibular stroke. In this study, regression models (for DHI and EQ-VAS at follow-up) clearly indicate that the degree of vertigo, dizziness, and imbalance in the acute stage of vestibular stroke has a significant impact on the long-term functional outcome and perceived health status (see Tables 2, 3). This finding is in accordance with a previous study, which indicated a worse outcome for patients with isolated cerebellar stroke, if they had more severe initial symptoms measured by the modified International Cooperative Ataxia Rating Scale (MICARS) (28). A relation between acute stroke symptoms and outcome is also well established for anterior circulation stroke (29–31). However, functional impairment and disability in posterior circulation stroke are not well represented in widely used stroke scales (such as the National Institutes of Health Stroke Scale—NIHSS), which may lead to some neglect for symptom severity in cerebellar and brain stem stroke. There is a need to establish and validate scales adapted to the posterior circulation to allow for a better assessment of HRQoL in patients with the chief complaints of vertigo, dizziness, imbalance, and double vision. Generally, the functional recovery after cerebellar and brain stem stroke is good in most cases both in our study and in literature (28, 32–35).

Outcome prediction cannot be based on quantitative testing of vestibular, ocular motor, or postural function only. While in acute vestibular stroke a moderate correlation is observed between the extent of spontaneous nystagmus and the severity of symptoms (8), many studies have failed to indicate a relationship between vestibular function tests (such as head impulse test and caloric irrigation) and subjective symptom severity in chronic vestibulopathies (9, 36). Similarly, in this study, vestibular test results assessed during the acute stroke stage (e.g., horizontal or vertical spontaneous nystagmus, and tilt of SVV) did not correlate with long-term functional outcome markers (data not shown). Given that vestibular signs in brain stem-cerebellar stroke tend to compensate rapidly (14) and completely (in 87% of patients in this study), it seems likely that the perceived symptoms and impairments are not related to vestibular processing only.

### Patient characteristics and traits as risk factors for worse outcome

In this study, anxiety trait, especially in combination with the female gender, was the most important patient-related predictor for functional outcome after vestibular stroke, while the effect of age was ambiguous (Tables 2, 3). Anxiety scores (STAI-T/STAI-S), depression scores (PHQ-9), vertigo-related symptom scores (DHI), and HRQoL scores (EQ-VAS) on follow-up showed a moderate to strong correlation in our study cohort. It is well established for various peripheral vestibular disorders that anxiety and depression have a significant impact on the symptom course and are associated with higher total DHI scores (6, 7, 37, 38). Anxiety and depression are closely related to secondary functional dizziness in vestibular disorders (7, 11, 12, 39, 40). On the contrary, anxiety and depression are negative predictors of functional recovery after stroke (41, 42). There are different potential explanation models for why anxiety may cause persistent dizziness following acute vestibular lesions: anxious patients tend to perceive vestibular symptoms and balance-related body sensations more intensely, which in turn leads to an unfavorable closed loop and voluntary motor control (including muscle co-contractions) (43). Anxiety-related avoidance behavior may result in reduced physical activity and consequently less sensorimotor adaptation to balance problems (5). This study is to our best knowledge the first to establish the impact of anxiety on functional outcomes also for patients with acute vestibular stroke.

Regression models in this study suggest a gender-sensitive impact of anxiety on the course of symptoms, i.e., anxiety and female gender had an additive effect on symptom persistence. On the contrary, gender as a single feature had no significant impact on outcome after vestibular stroke. The effect of gender in our study cohort seems to be mediated rather by accompanying anxiety traits. Epidemiological studies have shown that the prevalence of anxiety and depression in women is higher both in the general population and in patients with vestibular symptoms (44–46). Female patients seem to be more severely impaired by vestibular symptoms (36, 38, 47). Furthermore, women more often develop post-stroke depression. Therefore, female patients seem to be more vulnerable to enduring symptoms and perceived impairment mediated by anxiety and avoidance behavior following brain stem-cerebellar stroke, which is reflected by higher mean DHI scores as compared to men in our study (data not shown).

Patient age was not relevant for symptom severity at follow-up. This observation is in accordance with a study on isolated cerebellar strokes, which also found no age effect on the outcome (28). On the contrary, several studies on patients with anterior circulation stroke have shown worse outcomes (loss of independent living and mortality) with older age (29, 48). This seems reasonable at first sight, as older patients have more comorbidities, which may impair their recovery. The question remains if there is a basic difference in the compensation and recovery of vestibular symptoms (e.g., vertigo) and non-vestibular symptoms (e.g., hemiparesis). Previous studies suggested that older patients with vestibular disorders might recover to a similar extent as younger patients with a comparable vestibular lesion (49). However, other groups have documented some age-dependency of central vestibular compensation and plasticity (50, 51). One factor, which needs to be considered for this study cohort, is the mean age of 66 years. For supratentorial strokes, patients above the age of 70 years had persistent disability and worse outcome (52). Therefore, it remains an open question for further investigations, i.e., if very old patients will have an age-dependent decline in functional outcomes.

### Does stroke volume and localization predict outcome in vestibular stroke?

This study indicated stroke volume as a significant predictor of symptoms severity (Table 2). Previous studies were reporting controversial results about the impact of lesion size in posterior circulation strokes. Two previous studies also found stroke volume in cerebellar infarcts to be associated with outcome (34, 53). However, other studies failed to demonstrate a correlation between lesion size and persistent symptoms, but emphasized the importance of lesion localization and affected vascular territories for the latter (28, 33, 35). Ischemia in the SCA territory seems to cause more severe long-term impairment compared to PICA strokes, which is commonly explained by a higher degree of leg and gait ataxia in SCA strokes (28, 33, 54, 55). In the PICA territory, lesions in the posterior cerebellar lobe and around the dentate nucleus are associated with more persistent symptoms (35, 56). Furthermore, Baier and colleagues showed a clustering of impaired vestibular compensation of an SVV tilt with lesions of the lateral cerebellar hemispheres (lobule V, VI, VIIa) (57). For this study, we did neither find a difference of SCA vs. PICA strokes, nor of cerebellar vs. brain stem strokes, which most likely can be explained by the relatively small cohorts. For determination of the impact of lesion localization on functional outcome, more dedicated lesion-symptom mapping studies in larger cohorts of patients are needed.

### Strengths and limitations of the study

This prospective study included patient-related, symptom-related, and lesion-related factors to describe long-term disability and functional outcome of patients with vestibular stroke, instead of only evaluating quantitative neurophysiological parameters (such as SVV or spontaneous nystagmus). We think that this approach accounts more appropriately for the determinants involved in functional compensation and recovery after vestibular stroke. A limitation of this study is the variable timespan to follow-up. While all but two patients were followed >1 year after stroke, the exact timespan was not standardized. However, adjusting the regression models to the time of follow-up or excluding the two patients with a follow-up of <1 year after stroke did not change the results and relevant factors for the outcome (Supplementary Table 2). The main findings of this study remain stable, even though follow-up times varied within patients.

## Conclusion

The individual functional outcome in vestibular stroke mostly depends on the experience of acute symptom severity, unfavorable coping strategies for impairment by anxiety trait (especially in women), more extensive network damage by larger lesion volume, and older age. For the acute assessment of patients with vestibular stroke, commonly used stroke scales (such as the NIHSS or mRS) do not properly account for these factors. More meaningful scores and scales for the quantification of impairment and prediction of functional outcomes should be established in the future. Treatment plans should adapt the intensity of rehabilitation after vestibular stroke to the patients' risk factors. Psychological therapy elements should be considered in case of high anxiety traits.

## Data availability statement

The raw data supporting the conclusions of this article will be made available by the authors, without undue reservation.

## Ethics statement

The studies involving human participants were reviewed and approved by Ethics Committee of the University of Munich. The patients/participants provided their written informed consent to participate in this study.

## Author contributions

FS: study concept, collection of data, statistical analysis, data interpretation, and drafting of the manuscript. RS and EG: statistical analysis, data interpretation, and review of the manuscript. JC: imaging analysis, data interpretation, and review of the manuscript. KM and PJ: collection of data, data interpretation, and review of the manuscript. KJ: study concept, data interpretation, and review of the manuscript. MD: data interpretation and review of the manuscript. AZ: study planning, funding, data interpretation, drafting, and reviewing of the manuscript. All authors contributed to the article and approved the submitted version.

## Funding

This study was funded by the German Federal Ministry of Education and Research (BMBF) (Grant Number 01 EO 1401).

## Conflict of interest

The authors declare that the research was conducted in the absence of any commercial or financial relationships that could be construed as a potential conflict of interest.

## Publisher's note

All claims expressed in this article are solely those of the authors and do not necessarily represent those of their affiliated organizations, or those of the publisher, the editors and the reviewers. Any product that may be evaluated in this article, or claim that may be made by its manufacturer, is not guaranteed or endorsed by the publisher.
