# Supplementary material for: Determinants of functioning and health-related quality of life after vestibular stroke
Source: Front Neurol. 2022 Sep 8;13:957283. doi: 10.3389/fneur.2022.957283 (PMC9492892; doi:10.3389/fneur.2022.957283)
Supplement: Supplementary file 1 [file Table_1.DOCX]

|  | | | **n** | **percentage** |
| --- | --- | --- | --- | --- |
| **Stroke characteristics** | Chief complaint | Vertigo/Dizziness | 22 | 61.1% |
|  |  | Double vision | 6 | 16.7% |
|  |  | Both | 8 | 22.2% |
|  | Etiology | Arterio-arterial embolism | 16 | 44.4% |
|  |  | Cardial embolism | 11 | 30.6% |
|  |  | Unknown | 8 | 22.2% |
|  |  | Microangiopathy | 5 | 13.9% |
|  |  | Dissection | 2 | 5.6% |
|  | Vascular territories | PICA | 11 | 30.6% |
|  |  | SCA | 3 | 8.3% |
|  |  | Midbrain | 10 | 27.8% |
|  |  | Pons | 7 | 19.4% |
|  |  | Thalamus | 3 | 8.3% |
|  |  | Medulla | 2 | 5.6% |
|  | Therapy | Secondary prophylaxis only | 29 | 80.6% |
|  |  | i.v. thrombolysis | 3 | 8.3% |
|  |  | i.a. thrombectomy | 1 | 2.8% |
|  |  | Other | 3 | 8.3% |
| **Cardiovascular disorders and risk factors** | Cardiac disease | Atrial fibrillation | 11 | 30.6% |
|  |  | Coronary artery disease | 8 | 22.2% |
|  |  | Other cardiac arrthymia | 8 | 22.2% |
|  |  | Valvular abnormality | 7 | 19.4% |
|  |  | Myocardial infarction | 6 | 16.7% |
|  |  | Patent formen ovale | 5 | 13.9% |
|  |  | Heart failure | 3 | 8.3% |
|  |  | Other | 6 | 16.7% |
|  | Cardiovascular risk factors | Arterial hypertension | 23 | 63.9% |
|  |  | Hypercholesteriolemia | 22 | 61.1% |
|  |  | Family history of cardiovascular disease | 20 | 55.6% |
|  |  | Nicotine abuse | 19 | 52.8% |
|  |  | Rare/no sports activity | 14 | 38.9% |
|  |  | Previous stroke/TIA | 12 | 33.3% |
|  |  | Overweight | 10 | 27.8% |
|  |  | Diabetes | 6 | 16.7% |
| **Medication** |  | Statins | 27 | 75.0% |
|  |  | ASS | 25 | 69.4% |
|  |  | Beta blocker | 12 | 33.3% |
|  |  | NOACs | 10 | 27.8% |
|  |  | Insulin/blood glucose reducer | 5 | 13.9% |
|  |  | Vitamine K antagonist | 2 | 5.6% |

**Supplementary table 1:** Patient characteristics
