# Supplementary material for: Determinants of functioning and health-related quality of life after vestibular stroke
Source: Front Neurol. 2022 Sep 8;13:957283. doi: 10.3389/fneur.2022.957283 (PMC9492892; doi:10.3389/fneur.2022.957283)
Supplement: Supplementary file 2 [file Table_2.DOCX]

| **Model variables** | **Unstandardized Coefficients** | | **Standardized Coefficients** | **Significance level** |
| --- | --- | --- | --- | --- |
|  | B | Standard. Error | Beta |  |
| (Constant) | -93.012 | 35.58 |  | 0.017 |
| Sex | -5.79 | 8.59 | -0.11 | 0.509 |
| Age | 0.60 | 0.349 | 0.35 | 0.101 |
| STAI-T | 1.336 | 0.39 | 0.62 | 0.003 |
| Lesion volume | 0.94 | 0.44 | 0.33 | 0.045 |
| DHI acute stage | 0.49 | 0.18 | 0.52 | 0.013 |
| STAI-T_Sex | 2.35 | 0.78 | 0.55 | 0.007 |
| FU time | -0.10 | 0.33 | -0.05 | 0.754 |

**Supplementary Table 2:** Multiple linear regression model with DHI at follow-up as the dependent variable and adjustment for follow-up time. Lesion volume: in voxels devided by factor 1000; STAI-T_Sex: interaction variable of STAI-T and female sex; FU: follow-up; significance level <0.05.
